# Neurofeedback for opioid dependent patients in an outpatient setting: a pilot feasibility study

**DOI:** 10.1186/s13011-022-00458-2

**Published:** 2022-04-15

**Authors:** Helena A. Rempala, Justin A. Barterian

**Affiliations:** Department of Psychiatry and Behavioral Health, The Ohio State University – Wexner Medical Center, Columbus, Ohio USA

**Keywords:** Neurofeedback, Alpha/Theta Protocol, Feasibility, Opioid use disorder

## Abstract

**Background:**

Neurofeedback (NF) has been described as “probably efficacious” when used in conjunction with other interventions for substance use disorders, including the recent studies in the population of individuals with opioid use disorder. Despite these promising outcomes, the seriousness of the opioid epidemic, and the high rate of relapse even with the most effective medication-assisted maintenance treatments NF continues to be an under-researched treatment modality. This article explores factors that affected the feasibility of adding Alpha/Theta Neurofeedback to treatment as usual for opioid dependence in an outpatient urban treatment center. The study strived to replicate previous research completed in Iran that found benefits of NF for opioid dependence.

**Methods:**

Out of approximately two dozen patients eligible for Alpha/Theta NF, about 60% (*n* = 15) agreed to participate; however, only 2 participants completed treatment. The rates of enrollment in response to active treatment were monitored.

**Results:**

The 4 factors affecting feasibility were: (1) the time commitment required of participants and providers, (2) ineffectiveness of standard incentives to promote participation, (3) delayed effects of training, and (4) the challenges of researching treatment options not reimbursed by the insurance companies.

**Conclusions:**

The findings indicate that a large-scale study examining the use of NF for the treatment of opioid use disorder in the United States will likely be difficult to accomplish without modification to the traditional randomized control study approach and suggests challenges to the implementation of this treatment in an outpatient setting. A single-case methodology is proposed as a viable alternative.

## Background

Neurofeedback (NF) has been described as “probably efficacious” when used in conjunction with other interventions for substance use disorders (e.g., CBT; [1, 2]. The evidence was first documented in a population of individuals with alcohol use disorder [3, 4]; Saxby and Peniston [5]), then in polysubstance misuse [6, 7], opioid use disorder [8, 9], and crystal-methamphetamine use disorder [10]. Given these promising outcomes, the seriousness of the opioid epidemic, and the high rate of relapse even with the most effective medication-assisted maintenance treatments (e.g., methadone, naltrexone, and buprenorphine; [11]), one could expect that NF would become an actively researched treatment modality. One hypothesis regarding the paucity of research is that a NF study for opioid use disorder is not feasible due to several potential barriers (e.g., length of NF treatment, scarce availability of trained providers, financial barriers, delayed treatment effects, [12]. Indeed, typical protocols require up to 20 sessions, each lasting 30–40 min, occurring twice per week. Such length of training far exceeds the usual stay in an inpatient detoxification program (e.g., 3–7 days) and in any short-term residential treatment programs, typically 3–6 weeks long [11]. However, offering NF in an outpatient setting could provide a context where additional NF treatments could occur, minimizing the barrier to treatment created by short term inpatient and residential stays.

Only one study to date has examined the use of NF exclusively for opioid use disorder in an outpatient setting. Dehgani-Arani et.al. [9] conducted a randomized open-label study examining the use of the Scott-Kaiser modification of the Peniston protocol with 20 male participants diagnosed with opioid use disorder. Ten patients were randomized to NF plus treatment as usual (TAU) while 10 only received TAU. Participants were recruited from an outpatient substance use disorder clinic in Iran. Participants who received NF demonstrated significant improvement in the domains of somatic symptoms, depression, general mental health symptoms, and desire to use compared to the TAU group. Given the positive outcomes in this small yet successful study, the current study was designed to examine the feasibility of a randomized trial of NF for individuals with opioid use disorder in an outpatient setting in the United States.

## Methods

The study design was modeled after the Dehgani-Arani and colleagues’ clinical trial (2010, 2013). We selected a similar NF training protocol, expanded the inclusion criteria to include women, and selected the same measures to evaluate outcome variables. Consistent with the primary goal of evaluating the feasibility of NF in an outpatient setting, we added a measure of patient satisfaction. The eligible participants were randomly assigned to TAU or TAU and NF.

At the study onset, we planned to analyze the collected data by means of the generalized linear model (GLM, an extension of multivariate regression), that allows to combine the assignment to treatment variable (Treatment/Control) as well as the various covariates in the subject population. The possible set of covariates considered in the model included age, gender, SES, compliance with the treatment–as-usual and subjects’ length of substance use disorder. We expected the difference between the total scores on the Heroin Cravings Questionnaire (HCQ) pre- and post- treatment to be the primary outcome. To adjust for anticipated high variability in the subjects’ characteristics we expected to employ GLM with mixed effects, where some model coefficients may change from subject to subject. Two sample t-test or rank test would determine the statistical difference between the average change in the HCQ composite score for the Treatment and Control groups. We estimated that with the sample size of 24 subjects (12 in each treatment and control groups) should have a power of 70% to detect the difference of 1 standard deviation between groups at the alpha level of 5%. These numbers were arrived at with the help of the Power/Sample Size Calculator for Two Sample Inference retrieved from https://www.stat.ubc.ca/~rollin/stats/ssize/n2.html (see, for instance [13]). The secondary outcome variables were to be analyzed in a similar way. Given our intent-to-treat design, the data collected from all consented participants were to be analyzed.

### Participants

Inclusion criteria consisted of adults who were 25–45 years old, reporting at least 12 months of opioid dependence, and in the first 2–12 months of their recovery efforts. The exclusion criteria were seizures, traumatic brain injuries, migraines, and psychosis due to the potential the NF protocol may be contraindicated with these conditions All data collection and treatment occurred at a substance use disorder treatment facility in a large urban setting in Central Ohio. Treatment at this facility consists of three-day inpatient detox, several weeks of an Intensive Outpatient Program (IOP), Medication Assisted Treatment (MAT), and outpatient individual therapy with MAT for as long as therapeutically indicated. The participants were recruited from the existing patients who underwent extensive psychiatric assessments that included mental status examinations and differential diagnoses according to ICD10 classification of diseases. Given that the prospective participants had regular meetings with providers along the outpatient continuum (IOP to individual outpatient visits) at this facility, it was hypothesized that they would be able to consistently participate in NF.

### Measures

The feasibility of conducting NF training in the outpatient setting was assessed by collecting the rate of enrollment in response to active recruitment. Recruitment efforts consisted of providing information to providers during clinic meetings, weekly face-to-face orientation sessions for providers and patients in the IOP groups, and flyers and posters in the waiting room, group rooms and patient kitchen area.

In order to evaluate effectiveness, participants were asked to complete four assessment visits in addition to 30 NF sessions. At the research intake meeting, participants completed a clinical interview collecting basic demographic information, review of clinical records, history of presenting problem, and family history. They also completed a 15-min EEG Mini-mapping and three paper-and-pencil questionnaires: Heroine Craving Questionnaire (HCQ), Symptom Checklist 90-R (SCL-90-R) and Drug Abuse Screening Test (DAST). Random assignment to treatment condition (i.e., NF + TAU vs. TAU) took place at the end of the initial intake. The mini-mapping and the questionnaires were repeated at the end of training, 3- and 6- months later. At the end of treatment, participants completed the Client Satisfaction Questionnaire-Version 8 (CSQ-8).

EEG Mini-mapping was used to determine initial threshold values for the first 10 sessions of self-regulation training. The minimapping was performed according to the Institute of Applied Neuroscience guidelines (https://www.ian-asheville.com/about). EEG was recorded at the cranial site Fz with eyes open for two minutes, at the cranial site Cz with eyes open for two minutes, and at the cranial site Pz with eyes open for 2 min, closed for 2 min, and then reopened for 2 min. Average amplitudes on 5 frequencies channels collected during the 2-minute intervals served as summary scores. The five channels encompassed the following frequencies: 0–4 Hz (Delta), 4–8 Hz (Theta), 8–12 Hz (Alpha), 12–18 Hz (Beta), and 18–22 Hz (High Beta).

To assess desire to use, the Heroine Craving Questionnaire (HCQ) was used. It consists of 45 statements that respondents rate on a seven point Likert scale. It yields one total score and five subscale scores: (1) Anticipation of Positive Outcome; (2) Relief from Withdrawal; (3) Intention and Plan to Use Substance; (4) Desire to Use; and (5) Lack of Control Over Use. The HCQ has excellent reliability and validity and has been used in a number of studies evaluating opioid use disorder. The total score has demonstrated sensitivity to craving intensity [14].

The Drug Abuse Screening Test (DAST-10) was used to assess the degree of problems or consequences related to substance use. DAST-10 questions tap legal and family problems and medical issues. The DAST-10 has good internal consistency (0.92 for drug abuse and 0.74 for drug use). It correlates highly with the original longer version (*r* = .98; [15]).

Overall mental health was measured with the Symptom Checklist-90-Revised (SCL-90-R; [16]). The SCL-90-R is widely used in clinical and research settings. It consists of 90 patient-completed items that load on 11 scales, including: somatization, obsessive-compulsive tendencies, depression, anxiety, hostility, phobic anxiety, paranoid ideation, psychoticism, and overall psychopathology. The measure demonstrates acceptable reliability and validity. Individuals abusing substances demonstrate higher ratings on the following scales: obsessive- compulsive tendencies, anxiety, depression, and overall psychopathology [17].

Treatment acceptability was assessed using the Client Satisfaction Questionnaire-8 (CSQ-8). The CSQ-8 is an 8 item, self-report measure designed to assess satisfaction with healthcare treatments. The CSQ-8 demonstrates high internal consistency and correlates closely with termination status and severity of symptoms [18].

### NF training protocol

The neurofeedback training was administered using the hardware and software purchased form the EEGer company (eegerstore.com). The EEGer training system consists of several components: a set of sensors placed on top of the patient’s scalp; an amplifier that amplifies the electric signal collected by the sensors; a proprietary EEGer software that displays the signals and provides audio and/or visual feedback of the changes registered, a computer, and two screens; one screen to present the feedback as a set of measurements for the clinician (to monitor and guide the training) and second providing the same feedback in a form of a simple computer game with visual and/or auditory feedback for the patient to stay engaged in the process. In the present study the EEGer4 TM software was utilized [19].

Consistent with the Scott-Kaiser modification of the Peniston protocol, each participant was to originally receive 10 Sensory Motor Rhythm (SMR) training sessions for 10–20 min. The active electrode was placed at cortical site Cz with site A1 (i.e., left ear) used for reference and A2 (i.e., right ear) as ground. Increases in low beta (12–15 Hz) were reinforced while delta (2–5 Hz), theta (5–8 Hz), and high beta (18–30 Hz) were inhibited. Initial thresholds were set for participants to receive reinforcement for the low beta band 80% of the time, while keeping the other bands under the target threshold 20% of the time. If participants were able to keep the reinforcement band above the threshold 90% of the time for two trials, the thresholds were adjusted again in order to move closer to 80% reinforcement.

After SMR training, the participants began A/T training. Consistent with the Peniston protocol, A/T training occurred at cortical site Pz with site A1 as reference and A2 as ground for 20 min at a time. During the training, several bands were monitored including theta (5–8 Hz), alpha (8–12 Hz), beta (15–18 Hz) and delta (2–5 Hz). Depending on baseline waves, participants were initially instructed to lower their alpha levels below 12 mV, while increasing theta waves until alpha drops below theta, which is called “crossover.” After the bands “crossed over”, the participants were encouraged to increase both alpha and theta bands, while inhibiting delta. Participants heard independent sounds when moving theta and alpha in the desired direction, with the higher pitched sound representing alpha. At the beginning of each A/T session, the participants were led through 3- 5-minute blocks of guided imagery that included themes of engaging in behaviors to successfully abstain from using opioids and managing cravings. After each A/T training was completed, the participants were encouraged to discuss their experience.

## Results

### Feasibility

During the 8 months of active recruiting, the treatment facility received less than 24 new referrals to treat patients with opioid use disorder. A more precise number was unable to be obtained due to institutional data and privacy rules.

The study inclusion criteria needed to be modified several times in order to increase the pool of participants, given that the most interested patients did not meet the initial inclusion criteria. Table 1 summarizes the changes in the recruitment protocol in order to increase participation. The first major changes were implemented midway through the recruitment process. They consisted of abandoning randomization to a control TAU condition, which allowed all participants an opportunity to participate in the NF training, and the shortening the proposed length of self-regulation training from 10 to five sessions. The decision to shorten the number of sessions of the Scott-Kaiser modification was made after several prospective participants declined participation and the first few enrollees dropped out, almost all expressing concerns about the time commitment required for NF training. Shortening the modification instead of the A/T training seemed appropriate, because this adjustment has been proven particularly beneficial for patients “abusing stimulants” and with “attention-deficit EEG” [1, 10]. At the time we made the decision to shorten the training only one of the three participants was diagnosed with ADHD and had already dropped out from the study and not one of the prospective participants abused stimulants. It seemed therefore that, that the A/T protocol without the modification might still prove beneficial for our target population. We used the few sessions of the SMR training as an introduction to the neurofeedback training in general, ensuring that the subjects had a chance to train with their eyes open a few times, experience the visual and auditory effects of the regulatory EEG feedback, and experience their nascent ability to use the EEG feedback in the session. The change in the protocol was approved by the institutional review board (IRB). It was presented to participants as an attempt to shorten the training while still maximizing their exposure to the protocol that was designed to help their recovery from opioid use disorder. At the time the change was implemented, one of the participants had already completed 7 sessions of the SMR training. When the above changes did not produce an increase in recruitment, $10 gift card incentives were provided for participation after each NF session. Similarly, this change did not result in increased enrollment or completion of the protocol.

**Table 1:** Changes in the recruitment process over the 8 consecutive months

Design variables	October 2018	February 2019	March 2019
Study Design	Random assignment to TAU and TAU + NF^a^	Assignment to TAU + NF, no control (TAU only) condition	No change from 2/19
Proposed N	24	12	No change from 2/29
Inclusion Criteria	Males and females, 25–45 years old, at least 12 months of opioid dependency, in 2–12 months of recovery efforts	Males and females, 18 years and older, any length of dependency and any length of recovery	No change from 2/19
Exclusion Criteria	Seizures, traumatic brain injury, migraines	No change from 10/18	No change from 2/19
Proposed Intervention	10 Self-regulation sessions 20 A/T sessions scheduled twice a week	5 Self-regulation sessions 20 A/T sessions scheduled twice a week	No change from 2/19
Proposed Monetary Incentives for Participation in Research	$15 Gift Cards for each of 4 research assessments	No change from 10/18	No change form 2/19
Proposed Monetary Incentives for Participation in NF training	None	None	$10 for each attended NF training session
Number of Eligible Referrals	6 in 4 months	1 in 2 months	0 in 2 months

^a^*TAU *treatment as usual, *TAU + NF *treatment as usual plus neurofeedback

Despite eight months of recruitment efforts, only 15 patients agreed to be contacted by the research team and only ten of these patients were found eligible to participate upon the initial phone or face-to-face screening. Seven participants met inclusion criteria and enrolled in the study. Four of the participants were women and three were men. All self-identified as heterosexual. Participants were White (*n* = 4) and African American (*n* = 3). Ages ranged from 33 to 65 years. All participants traced the onset of their opioid use disorder to a prescribed pain relief therapy. On average, they were dependent on opioids for 15 years (range: 2–44 years) and tried to quit about four times in their lives (range: 2–6 years). At the time of the subjects’ research intake, their length of sobriety varied greatly, from two days to 2007 days. All participants were diagnosed with Severe Opioid Dependence with Withdrawals. Five participants also carried additional psychiatric diagnoses such as Major Depressive Disorder (*n* = 2), PTSD (*n* = 1), Bipolar Disorder (*n* = 1), Generalized Anxiety Disorder (*n* = 1), Attention Deficit Hyperactivity Disorder (*n* = 1), and Obsessive Compulsive Disorder (*n* = 1).

Of the seven participants enrolled in the study, three declined further participation after the first incentivized research assessment. Two participants dropped out after a handful of self-regulation training sessions (5 and 1) and before the A/T protocol was implemented. Out of the remaining two participants, one completed 23 sessions of NF training (3 self-regulation and 20 A/T) and the second participant completed 25 sessions of NF training (7 self-regulation and 18 A/T). Those same two participants also completed all but one of the 4 research appointments.

### Training effects

**Fig. 1:**
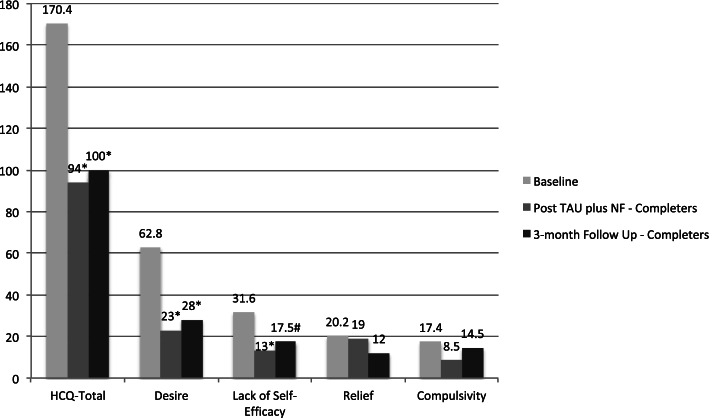
Heroine Cravings Questionaire Total and the subscale mean baseline scores for non-completers compared to the mean scores of completers at the end of training and at 3-months follow up. * *p* < .05, #*p* < .10

Pre- and post-test scores of the two participants who completed the NF protocol suggest that NF + TAU resulted in improvements on measured symptoms. The Exact Statistical Test was used to examine how likely the observed improvement might have occurred by chance alone for completers (*n* = 2) and non-completers (*n* = 5) using mean scores. This comparison was conducted in two steps by means of the Fisher-Pitman permutation test [20], which is a method of statistical analysis in small sample sizes. In the first step, it was established that the mean pre-test scores in both groups were not statistically different (*p* > .10). In the second step, the mean post-test scores of the completers were compared with the pre-test scores of the non-completers. The t-test statistic was used with the permutation distribution obtained by randomly re-labeling the scores of completers and non-completers in order to establish the statistical significance of the observed differences. Given the sample sizes, no multiple comparison adjustments were made when calculating the statistical significance. Those unadjusted comparisons suggested significant (*p* < .05) improvement for those who completed the protocol on the Heroine Cravings Questionnaire at the end of treatment. At three months follow up, the trend remained with the completers showing improvement on the Desire to Use (*p* < .05) and an improvement trend on the Lack of Self-Efficacy (*p* < .10) subscales over time (See Fig. 1).

**Fig. 2:**
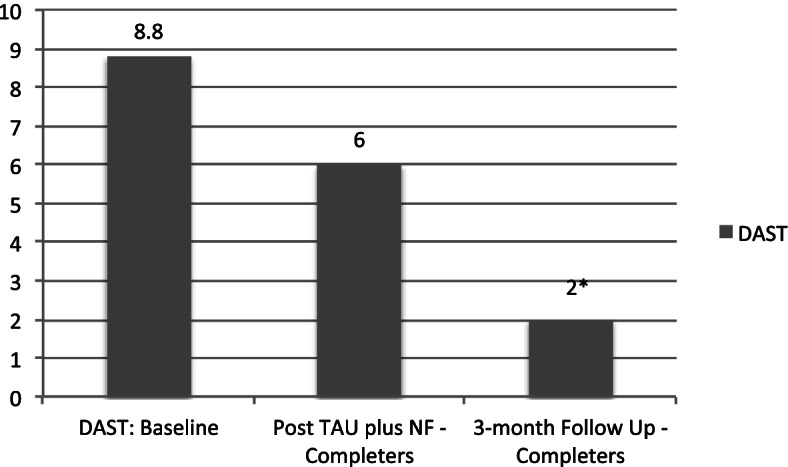
Drug Abuse Screening Test mean baseline scores for non-completers compared to the mean scores of completers at the end of training and at 3-months follow up. * *p* < .05

The comparison of the DAST score yields a trend (*p* < .10) suggesting diminished negative consequences from using drugs right after the completion of TAU + NF in comparison to the consequences reported at baseline. The trend became statistically significant (*p* < .05) three months after the completion of TAU plus NF (See Fig. 2).

**Fig. 3:**
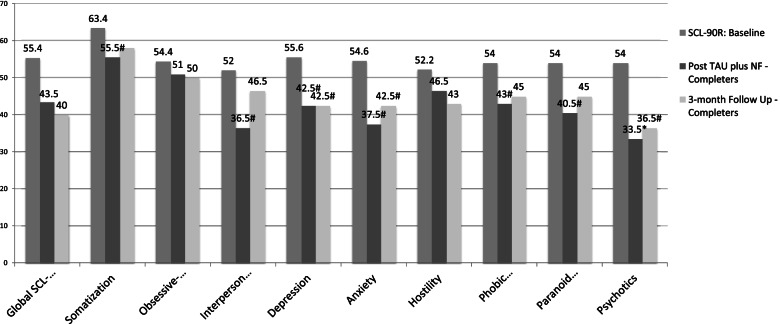
Symptom Checklist-90-Revised Global and subscale mean baseline scores for non-completers compared to the mean scores of completers at the end of training and at 3-months follow up. * *p* < .05, #*p* < .10

Finally, participants who completed NF + TAU had a trend of improved post-treatment scores on 6 of the 9 subscales when compared to non-completers (Somatization, Interpersonal Sensitivity, Depression, Anxiety, Phobia, Paranoia, *p* < .10) and a significant improvement on the Psychoticism subscale (*p* < .05) The observed trends were still visible on the Depression, Anxiety and Psychoticism subscales 3 months after treatment was completed (See Fig. 3).

## Discussion

The failure to recruit and retain patients for NF + TAU underscores the barriers associated with implementing NF treatment and the complexity of researching NF treatments within this patient population. These barriers include the length and intensity of NF treatment as well as a delay in treatment effects. Additionally, barriers related specifically to researching NF with an opioid use disorder population include difficulty with treatment completion as well as complying with research related timelines.

### Length and intensity of treatment

Only a small percentage of our sample (about 20%) was ready to commit extra time to NF training, especially after learning that the training does not guarantee treatment success. Our participants cited childcare, court appearances, school and work schedules, and other therapies (for example, physical therapy appointments), as reasons for their lack of time to spare for the research activities.

True to the state of science, the researchers presented NF as a relatively novel approach with promising but less than certain results. It is possible that presenting NF as an elective within IOP that would replace up to 1 h of the required TAU component may result in more patients being willing to try it. However, this approach is potentially problematic as it could be seen as unethical given that NF would be replacing a potentially more effective evidence-based treatment. Further, the lack of insurance reimbursement for NF would likely require the outpatient program to reduce its revenue by one hour spent on NF training. In an inpatient or a residential program more patients may choose to participate in additional activity in their free time to maximize their chances of success.

### Delayed treatment effects

The positive effects of NF were not detected by the participants immediately after training. While this is a common problem for most mental health treatment modalities, it affected NF compliance just as it affects other modalities. It is possible that the outpatient population would be more willing to give NF a chance if the initial self-regulation sessions were spread out over more time (to minimize the inconvenience) and were individualized (possibly using the z-score training) to optimize level of thresholds, cranial site, and direction of training for each patient. Not surprisingly, our two completers actually experienced relief from sleep disturbances and anxiety within just a few self-regulation sessions. They attributed the improvement in those symptoms to NF training and their motivation for participation strengthened. At the end of training they reported high levels of satisfaction from the training despite its length and intensity. Moreover, they seemed to have maintained some degree of gains in their overall wellbeing up to 3 months later.

### Participant incentives

As reported in Table 1, incentivizing treatment participation beyond the intake assessment did not produce any positive referrals. Moreover, the two participants that finished NF training completed a significant number of their training sessions before the incentives were introduced. Thus their motivation to participate seemed minimally influenced by being reimbursed for their time. Another participant was informed of additional incentives and expressed interest but did not follow through with coming to the appointments. Four other participants only received incentives for their first research appointment and were not willing or able to commit to anymore activities that required extra scheduling. Overall, treatment incentives were not successful in encouraging participation or treatment completion.

### Timeline constraints

The final barrier to NF research, complying to research timelines, is pertinent to the feasibility of time-limited clinical trials in the NF field of study. The time requirement placed on this research turned out to be unrealistic for the chosen population and the studied intervention (A/T training). Our experience suggests that imposing deadlines on NF researchers by the funding agencies may contribute to the limited sample sizes. Likewise, lack of insurance reimbursement for NF makes the non-funded research particularly challenging. Further, the length of time required to collect an eligible sample of participants introduces a serious time confound into the analyses. Thirty years into its existence, the field seems forced to advance using small N and single-case study designs, especially in this population.

## Conclusions

Our experience brings to the forefront challenges hampering advances in understanding the efficacy of NF in substance abusing populations. Due to the lack of efficacy studies, patients may be reluctant to participate in exploratory or time-consuming research. However, without their participation, no large clinical trials can be conducted. The feasible small N designs and single-case studies provide evidence often judged as insufficient to add NF interventions to the readily available and reimbursable treatment options in the U.S.A. Of importance, the same lack of “hard” evidence encourages the continuous use of NF in more affluent populations despite its potential ineffectiveness.

One option for the NF researcher interested in challenging populations would be to seek funds to conduct large, multisite randomized clinical trials, similar to the one secured by ADHD researchers [21]. The large recruitment areas may allow for the collection of adequate sample sizes within a reasonable amount of time. The other option would be to create a standard research protocol for pre- and post- NF assessment and impose one standard of coordinated care by NF providers. This “combining of forces” approach would allow the NF scientists to improve the reliability and validity of their efforts and, even more importantly, to create a repository of patient’s raw data available for analysis. Large sample sizes would allow for statistical control of the time confound rendering future analyses more accurate.

Finally, if the feasibility of large N studies using Neurofeedback, especially with vulnerable populations, is seriously called into question, then a paradigm shift toward the methodology of series of single-case designs seems like the most logical next step in the scientific scrutiny. Not only are the single-case designs less costly and easier to implement with vulnerable populations than the large N studies, but they also allow for a case-by-case analysis of the circumstances under which the NF training works best. In addition, as the proponents of this approach argue, the individualized data will inform us about what “mechanisms are responsible for patients’ ability to respond to neurofeedback” an issue at the forefront of the recent NF efficacies studies [22].

## Data Availability

The datasets used and/or analyzed during the current study are available from the corresponding author on reasonable request,

## Abbreviations

A/T: Alpha/Theta
CSQ-8: Client Satisfaction Questionnaire-8
DAST: Drug Abuse Screening Test
HCQ: Heroine Craving Questionnaire
IOP: Intensive Outpatient Program
MAT: Medication Assisted Treatment
NF: Neurofeedback
SCL-90-R: Symptom Checklist 90-Revised
SMR: Sensory Motor Rhythm
TAU: Treatment as usual
